# Formulation and Characterization of Nicotine Microemulsion-Loaded Fast-Dissolving Films for Smoking Cessation

**DOI:** 10.3390/molecules27103166

**Published:** 2022-05-16

**Authors:** Kantaporn Kheawfu, Pattaraporn Panraksa, Pensak Jantrawut

**Affiliations:** Department of Pharmaceutical Sciences, Faculty of Pharmacy, Chiang Mai University, Chiang Mai 50200, Thailand; kantaporn.kheawfu@cmu.ac.th (K.K.); pattaraporn.prs@gmail.com (P.P.)

**Keywords:** nicotine, microemulsion, fast-dissolving film, buccal film

## Abstract

The present study aimed to develop a nicotine microemulsion (NCT-ME) and incorporate it into a fast-dissolving film. The NCT-ME was prepared by mixing the specified proportions of nicotine (NCT), surfactant, co-solvent, and water. The NCT-ME was measured by its average droplet size, size distribution, zeta potential, and morphology. NCT-ME fast-dissolving films were prepared by the solvent casting technique. The films were characterized by morphology, weight, thickness, disintegration time, and mechanical strength properties and the determined NCT loading efficiency and in vitro drug release. The results showed that almost all NCT-MEs presented droplet sizes of less than 100 nm with a spherical form, narrow size distribution, and zeta potentials of −10.6 to −73.7 mV. There was no difference in weight and thickness between all NCT-ME films, but significant changes in the disintegration times were noticed in NCT40-Smix[PEG-40H(2:1)]10 film. The mechanical properties of films varied with changes in type of surfactant. About 80% of the drug release was observed to be between 3 and 30 min. The drug release kinetics were fitted with the Higuchi matrix model. The NCT40-Smix[P-80(1:1)]10 film showed the highest dissolution rate. It was concluded that the developed ME-loaded fast-dissolving film can increase drug release to a greater extent than the films without ME.

## 1. Introduction

Aggressive stop smoking campaigns in Thailand have continued to increase in frequency. According to the National Statistical Office data, it was found that the number of smokers in Thailand was reduced from 25.47 to 19.90% during 2011–2015 [1]. However, stopping smoking suddenly can lead to withdrawal symptoms from the nicotine in cigarettes that causes a strong smoking urge, irritability, and insomnia. These symptoms could occur for 2–12 h [2], but they can be reduced by nicotine replacement therapy (NRT). NRT via oral administration is limited due to the rapid destruction of nicotine by the liver via the first-pass metabolism [3]. Therefore, other forms of nicotine (NCT) delivery systems have been developed to reduce the metabolism of this drug in the body, including gum, lozenges, mouth spray, oral films, and transdermal patches.

Each drug delivery form has advantages and disadvantages. The transdermal patch form has a slow, long-lasting release of NCT, which is different from fast-acting cigarettes. A common side effect of the transdermal patch is itching or redness in the area of skin which is in contact with the patch [4]. In contrast to this NRT, the NCT gum is permeated through the buccal mucosa, and it is convenient to use and carry. The clinical guidelines for treating tobacco addiction reveal that NCT gum and high-dose topical NCT patches are more effective in quitting smoking than taking a placebo. It was found that the two forms of smoking cessation aid were not different in effectiveness [5]. Despite the advantages of NCT gum, it has a complex approach causing a relatively high incidence of incorrect use. Chewing without control of NCT release can cause large amounts of NCT to accumulate in the saliva. The saliva containing the NCT tends to be swallowed down the gastrointestinal tract and not absorbed via the buccal mucosa, which is not aligned to the purpose of chewing NCT gum. As a result, the NCT is not received in the correct dose for smoking cessation and irritates the gastrointestinal tract [6].

In order to avoid the risk of NCT gum, it is an interesting option to use a buccal film that is intended to deliver the drugs that need to be absorbed through the buccal, which is similar to NCT gum behavior. The buccal film is easy to use and does not require chewing, which can often cause the extreme variation of NCT adsorption. The film would adhere to the buccal mucosa and dissolve rapidly. This allows the drug particles to be released from the film and then diffuse through the buccal tissue to capillaries. Consequently, the drug is absorbed directly into the bloodstream without damage by the liver and increases the drug bioavailability [7].

The important component of buccal film is the type of polymer and the most commonly used are gelatin, pectin, carboxymethyl cellulose, and hydroxypropyl methylcellulose (HPMC). HPMC is a nonionic polymer having a linear glucose molecule structure and it is stabilized by hydrogen bonds. The methyl substitution of HPMC is performed using the substitution of free hydroxyl groups of glucose with hydroxypropyl groups, in order to enhance the cellulose backbone’s viscosity, solubility, gelation, and film-forming ability. Other advantages of HPMC are that it is a stable, odorless, flavorless, and edible substance with excellent film-forming characteristics. As a result, HPMC polymer may be utilized in a variety of applications, including drug delivery [8]. Okeke et al. developed a NCT buccal film containing HPMC, sodium alginate, and glycerin. Magnesium aluminum silicate as a nicotine stabilizer and its content were compared at different concentrations in the research. The results showed that the magnesium aluminum silicate had an effect on swelling and resulted in a slower release of NCT from the film [9]. Khan et al. prepared a fast-dissolving oral film consisting of HPMC, glycerin, and polyoxyethylene glycols-400 (PEG-400) at various concentrations by the solvent casting method. The study focused on the appearance, thickness, mechanical properties, film-breaking time, and NCT release from the film. It was found that films containing HPMC and PEG-400 showed effective characteristics. The results also showed up to 20% of the drug can be released within 2 min, and the total drug release can be done in 20 min [10].

In addition, the buccal film can be incorporated with microemulsion (ME) systems to enhance the drug loading efficacy and drug dissolution rate. ME consists of water, oil, surfactant, and a co-surfactant or co-solvent that can be spontaneously generated without energy, which is different from conventional emulsion systems. Moreover, the ME also has small internal particles with a large surface area, which allows the drug to be more soluble and absorbed [11]. Therefore, the hypothesis is that if the ME system was applied into the film, it would increase the permeability of the drug effectively. This research aimed to develop a nicotine microemulsion (NCT-ME) and incorporate it into a buccal film using hydroxypropyl methylcellulose with a low-viscosity grade (HPMC E15) as a film-forming agent, for ease of use and efficacy as an alternative NRT, which will hopefully increase the success rate of smoking cessation.

## 2. Results and Discussion

### 2.1. Development and Characterization of NCT-ME

The results showed that the obtained 24 NCT-ME formulations were light brown in color and clear, without phase separation. The mean droplet size of the NCT-ME is shown in Table 1. Smix was a mixture of surfactant and polyethylene glycol-400 (PEG-400) as a co-surfactant at various weight ratio. Almost all of the NCT-ME presented mean droplet sizes of less than 100 nm, whereas NCT50-Smix0 and N40-Smix[P-80(1:2)]10 showed a significantly (*p* < 0.05) larger mean droplet size (1237 and 215.8 nm, respectively). NCT50-Smix0 is a surfactant- and co-surfactant-free formulation, resulting in the largest particle size and the highest polydispersity index (PDI) value (0.73), indicating the board distribution of the particle size. The zeta potential of all NCT-ME was in the range of −10.6 to −73.7 mV. The highest zeta potential (−73.7 mV) represents the charged surface of the NCT drug particles without the surrounding non-ionic surfactant. NCT has a weak base chemical composition and be formed of negatively charged ions [12]. The lower zeta potential value of NCT-ME, except NCT50-Smix0, might be attributed to the hydrogen bonding between the pyrrolidine site of NCT and the nonionic surfactant [13].

The different compositions of NCT-MEs presented no significant difference in zeta potential. The type of surfactant had no effect on zeta potential since both P-80 and PEG-40H are nonionic surfactants, which may change the charge behavior when combined with an ionic surfactant [14,15]. The various compositions of NCT and Smix, as well as the Smix ratio, did not affect zeta potential in the present study. Moghimipour et al. indicated that the composition of oil and Smix had no effect on zeta potential, but the Smix ratio of the microemulsion formulation using nonionic surfactants had a more positive effect on the zeta potential. The study compared the Smix ratio in a ratio of 6:1 and 4:1. A higher ratio of surfactant than co-surfactant (6:1) would result in a positively charged composition, from the reverse hexagonal and micellar structures, while a 4:1 ratio produces micellar and bicontinuous structures [16]. However, the present study compared the Smix ratio in a ratio of 2:1, 1:1, and 1:2, which may not be sufficient to see the effect of the Smix ratio on the zeta potential.

NCT-ME was be incorporated into HPMC films in the subsequent study. The pure HPMC solution had a zeta potential value of −2.14 to −3.4 mV. After the NCT-ME with the negative charge was incorporated into the HPMC film, this can increase the electric charge of NCT particles and increase the repulsive forces between particles, resulting in a uniform distribution of the particles in the HPMC film. Therefore, HPMC can stabilize NCT molecules in its matrix and prevent the accumulation of NCT molecules on the surface of the film [17].

The effect of the proportion between NCT and Smix in the formulation had no effect on ME formulation except for the formula of NCT40-Smix[P-80(1:2)]10, which contained 40% NCT and 10% Smix that contained P-80 and PEG-400 at the ratio of 1:2 yielded mean droplet size at 215.8 nm with a PDI value of 0.34. NCT40-Smix[P-80(1:2)]10 had a higher content of co-surfactant than surfactant; however, the formula of NCT40-Smix[PEG-40H(1:2)]10 using the surfactant PEG-40H showed different results in mean droplet size at 21.7 nm with a PDI value of 0.26, indicating that the ratio between surfactant and co-surfactant does not affect such surfactant.

The polyoxyethylene nonionic surfactants, P-80 and PEG-40H have hydrophilic–lipophilic balance (HLB) values of nearly 14–16. P-80 is polyoxyethylene sorbitan oleate whereas PEG-40H is a polyoxyethylene castor oil derivative. Both surfactants are often used in ME due to their alkyl chain having a positive effect on the penetration of oil onto the curved film interface [18,19]. Surfactant can assist to enhance microemulsion production by reducing the interfacial tension and forming an interfacial layer. The positioning of surfactant molecules at the oil–water interface decreases the free energy required to form the emulsion, resulting in improved oil droplet stability [20].

The results presented in Table 1 also demonstrated that surfactant and co-surfactant impacted the droplet size and PDI value. The ratio between surfactant and co-surfactant and the type of surfactant did not affect other microemulsions. Therefore, NCT-ME formulations containing the most NCT and yield particle sizes less than 100 nm, with narrow PDI values, were chosen. The ME containing both P-80 and PEG-40H yielded the same zeta potential results. However, these NCT-ME formulations are dispersed in the polymer solution once they are loaded into a film. It was assumed that the effect of zeta potential may not influence the tendency of stability. The selected microemulsions were NCT40-Smix[PEG-40H(1:2)]10, NCT40-Smix[P-80(1:1)]10, NCT40-Smix[PEG-40H(1:1)]10, NCT40-Smix[P-80(2:1)]10, and NCT40-Smix[PEG-40H(2:1)]10, which contain the same amount of NCT and Smix in all formulas, were 40 and 10%, respectively, and NCT50-Smix0 was used as a control.

**Figure 1:**
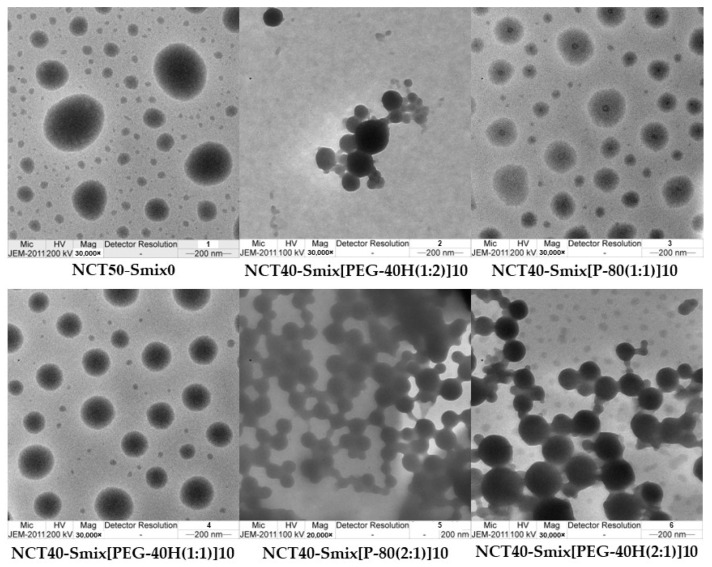
TEM images of NCT50-Smix0 and selected NCT-ME after 100-fold dilution with water; scale bar: 200 nm.

The internal droplet morphology of 100-fold diluted NCT-ME formulations that were examined using transmission electron microscopy (TEM) presented small droplet sizes. The droplets appeared to be of a spherical form (Figure 1). The average size determined from the scale bar was around 200 nm, showing a nanosized range similar to those reported by dynamic light scattering (DLS). The largest droplet sizes were observed in NCT50-Smix0 without surfactant, which was around 500 nm.

### 2.2. NCT Fast-Dissolving Films

**Figure 2:**
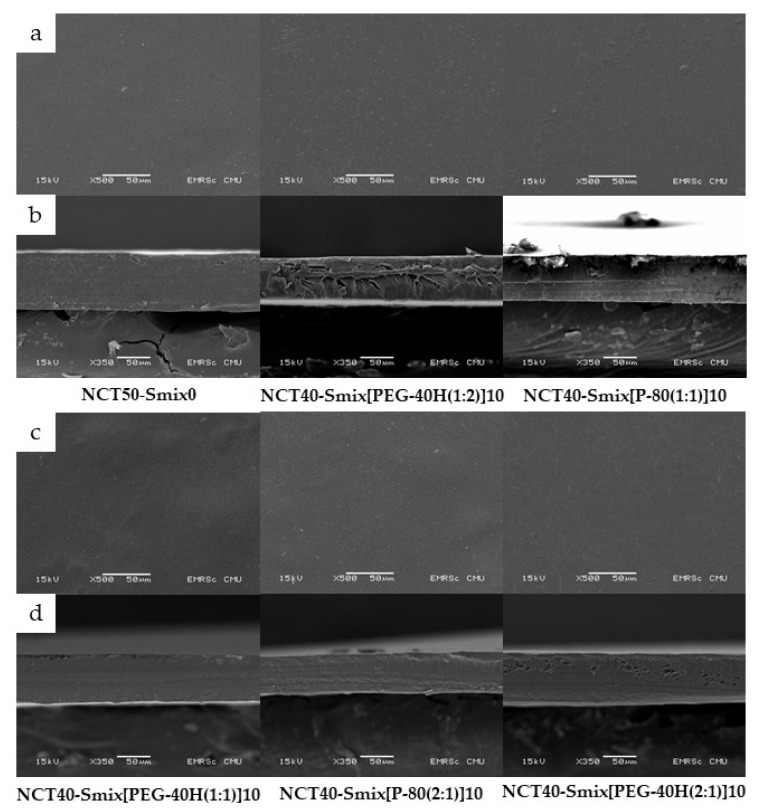
Scanning electron microscopy (SEM) images of NCT-ME fast-dissolving films, at the surface (**a**,**c**) and cross-sections (**b**,**d**), at 500× and 350× magnifications, respectively.

The generated NCT-ME fast-dissolving films by the solvent casting process had a transparent appearance with a light brown film. The light brown color was originally from NCT. Figure 2 shows scanning electron microscopy images of films. The NCT-ME fast-dissolving films had a homogenous and continuous morphology, showing that NCT was distributed uniformly throughout the film’s formulation. The NCT-ME fast-dissolving films with thicknesses ranging from 73 to 85 μm, as presented in Table 2, were related to the SEM images. The thickness of the film depends on the amount of solution injected into the dish during the casting process.

The result of thickness, weight, disintegration time, and normalized disintegration time of NCT-ME fast-dissolving films is shown in Table 2. The thickness of the NCT-ME film measured with a micrometer equaled the thickness determined by scanning electron microscopy (SEM), as presented in Figure 2. Between all NCT-ME and NCT50-Smix0 films, there was no difference in thickness or weight. The normalized disintegration time is the disintegration time of each film divided by the ratio between its thickness, and the minimum values of an average thickness of all formulations. This value is calculated to reduce film variation. However, there was a difference in disintegration time and normalized disintegration time between NCT40-Smix[PEG-40H(2:1)]10 films and the other films (*p* < 0.05). It was noticed that the NCT40-Smix[PEG-40H(2:1)]10 film had lower normalized tensile strength and percent elongation at breaking than the NCT50-Smix0 film and some NCT-ME fast-dissolving films, as shown in Table 2, which led to the shortest disintegration time. The disintegration time and normalized disintegration time of the NCT-ME fast-dissolving films were both less than 32 s, which is an acceptable disintegration time for a fast-dissolving film, which is defined as a film that disintegrates or dissolves in less than 60 s after being placed in the mouth without drinking water or chewing [21].

The results of mechanical properties and NCT loading efficiency are shown in Table 3. Smix that was incorporated into the film significantly influenced normalized the tensile strength, percent elongation at break, and Young’s modulus values. In addition, some Smix decreased normalized tensile strength and percent elongation at break, whereas some of them could alternate Young’s modulus values. The normalized tensile strength is the tensile strength of each film divided by the ratio between its thickness and the minimum values of an average thickness of all formulations. An ideal buccal film should have a sufficiently high tensile strength value and high Young’s modulus values to endure regular handling. Although, a very high value is not desirable since it may slowly induce drug release from the polymer matrix [22]. NCT-ME fast-dissolving films exhibited very high NCT loading contents. NCT40-Smix[P-80(1:1)]10 film and NCT40-Smix[P-80(2:1)]10 film using P-80 as the surfactant appeared to have higher NCT loading contents than PEG-40H formulations. NCT40-Smix[PEG-40H(1:1)]10 film presented the lowest NCT loading contents, therefore, this film was excluded from further study.

Elshafeey et al. developed and optimized palatable oral fast-dissolving films loaded with a paroxetine nanosuspension using P-80 as a wetting agent. It was found that incorporating Tween 80 into the film can increase drug permeation. Since the surfactant created an elastic function that loosened or fluidized the mucosal membrane lipid bilayer and allowed the drug to incorporate into deeper layers of the biological membrane, leading to an increase in the drug permeability through the buccal mucosa [23]. No previous study used PEG-40H in oral film formulations, however, the recent study resulted that PEG-40H yielded a film effect that was similar to that of using P-80 as a surfactant.

### 2.3. In Vitro Release Study of NCT-ME Fast-Dissolving Films

**Figure 3:**
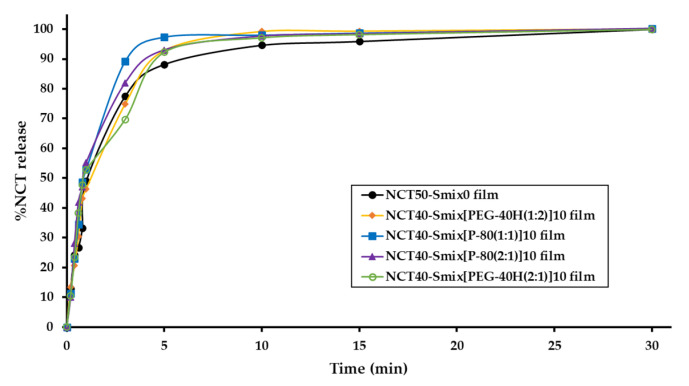
In vitro drug release profile of NCT-ME fast-dissolving films.

The dissolution studies were carried out under the sink condition. A medium volume of at least three times higher than that required to generate a saturated solution of the drug substance is referred to as sink conditions. The accumulated concentration of sample in the dissolution medium (φ) is less than one-third of its saturation solubility [24]. On the other hand, NCT is soluble in water at temperatures below 60.8 °C (1 × 10^6^ g/L); consequently, the calculated φ value is 0.0001 and was less than one-third of its saturation solubility [25,26]. As a result, this in vitro release experiment was classified as a sink condition. As illustrated in Figure 3, the in vitro drug release profile of NCT-ME fast-dissolving films is displayed as a plot, representing a correlation between NCT release percentage and time. The t_80%_ of NCT release was observed between 3 and 30 min in films. The average release of NCT at the end of the experiment (30 min) was 100.0 ± 0.1%. The NCT40-Smix[P-80(1:1)]10 film had the fastest dissolution rate than other films during 3–10 min.

Modeling release kinetics using power law equations is simple and may assist in the elucidation and explanation of transport mechanisms. The release mechanism of NCT from NCT-ME fast-dissolving films was investigated using four mathematical models, as shown in Table 4, including the zero-order model, the first-order model, Higuchi model, and Korsmeyer–Peppas empirical power law. Based on the value of the correlation coefficient (*r^2^*) obtained from the linear regression analysis, relevant mathematical models can be selected according to each model. The results showed that the Higuchi model best described the release behavior of NCT from all NCT-ME fast-dissolving films, implying that the drug release mechanism was diffusion from polymeric systems.

Among all five formulations, the “k_H_” value was the highest for NCT40-Smix[P-80(1:1)]10 film (60.602). The Higuchi model of all the formulations showed higher correlation coefficient values (0.862–0.975), indicating diffusion as the release mechanism. Based on the above results, the NCT40-Smix[P-80(1:1)]10 film showed the highest dissolution rate and lowest normalized tensile strength and Young’s modulus values, being appropriate for NCT-ME fast-dissolving films. The significant differences in Young’s modulus value of the NCT40-Smix[P-80(1:1)]10 film describe film rigidity, which might affect the film’s wettability, water penetration into the polymer network, and alterations in its dissolution rate. According to the redispersed particle size results, it seems interesting to note that the particle size of NCT-ME may not have affected the dissolving rate because all films had similar particle sizes, except the NCT50-Smix0 formulation. It was noticed that films with P-80 showed a better dissolution relative to higher drug loads, and this could be the probable reason for this dissolution enhancement. Therefore, it is reasonable to speculate that differences in dissolution rate are mostly due to the surfactant type used in the NCT-ME fast-dissolving films. The results illustrated that the developed films with ME can increase the dissolution rate.

## 3. Materials and Methods

### 3.1. Materials

Absolute ethanol, methanol (HPLC grade), glacial acetic acid, and sodium acetate were purchased from RCI Labscan Limited (Bangkok, Thailand). Polysorbate 80 (P-80), polyethylene glycol-40 hydrogenated castor oil (PEG-40H), and polyethylene glycol-400 (PEG-400) were purchased from Sigma-Aldrich (Darmstadt, Germany). Trimethylamine and phosphoric acid were purchased from Loba Chemie Pvt, Ltd. (Mumbai, India). Analytical standard nicotine (NCT), with a purity of 98.9%, was purchased from Sigma-Aldrich (St. Louis, MO, USA). Hydroxypropyl methylcellulose E15; HPMC E15 (AnyCoat^®^-C AN15, substitution type 2910, viscosity 15 mPa·s) was purchased from Lotte Fine Chemical Co., Ltd. (Seoul, Korea).

### 3.2. Preparation and Characterization of NCT-ME

#### 3.2.1. NCT-ME Preparation

**Figure 4:**
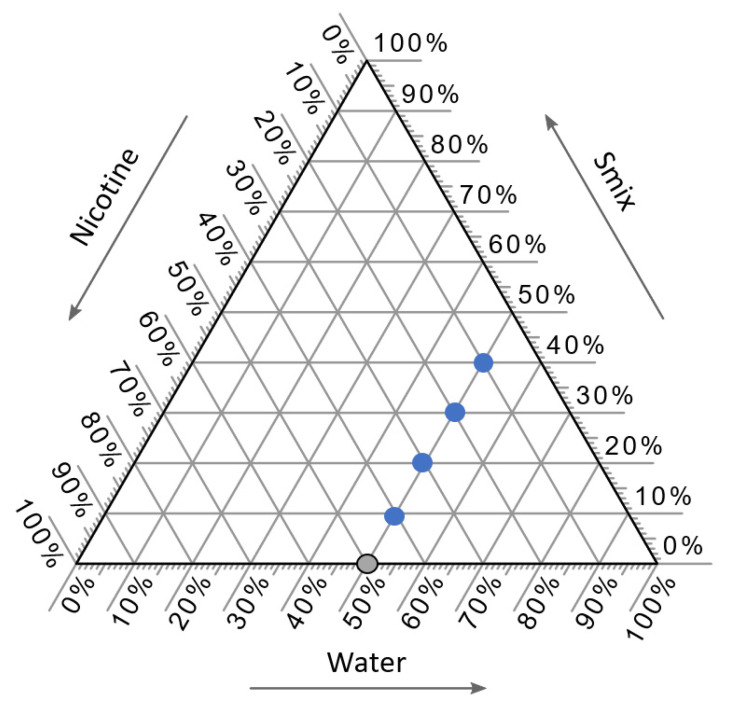
Selected components from pseudo-ternary phase diagrams of the NCT-ME, which were composed of NCT, Smix, and water. The blue circles represent the NCT-ME formulations, whereas a gray circle represents the NCT without ME.

Five formulations were selected with a fixed quantity of water, which was set at 50% *w*/*w* using a pseudo-ternary phase diagram, as presented in Figure 4. The water content was fixed at 50% *w*/*w* since the high water content can be aided with film formulation. One gram of each NCT-ME formulation was prepared by mixing the specified proportions of NCT, Smix (mixture of surfactant and co-solvent), and water, as shown in Table 5. The inclusion of the two surfactants P-80 and PEG-40H, and the co-surfactant PEG-400, was due to their high levels of miscibility with NCT. Smix were prepared using the weight ratios of 1:2, 1:1, and 2:1. Formulations were made with NCT-ME preparations containing 10–50% NCT; 3.33–26.67% P-80 or PEG-40H; 3.33–26.67% PEG-400; the water content fixed at 50%. Twenty-four NCT-ME formulations were devised. The names of these NCT-ME formulations were made using the percentage of NCT, the type of surfactant in Smix (Smix[P-80] or Smix[PEG-40H]), and the Smix weight ratio, followed by the number representing the percentage of their compositions. For example, NCT10-Smix[P-80(1:2)]40 represents an NCT-ME formulation with 10% of NCT, 40% Smix in the surfactant (P-80) and the co-solvent (PEG-400) with a weight ratio of 1:2, and 50% water (which is fixed at this ratio). The component concentrations that yielded transparent mixtures lasting for at least 24 h were recognized as ME.

#### 3.2.2. Characterization of NCT-ME

The average droplet size and size distribution of the obtained NCT-MEs were measured by DLS using a Zetasizer Nano (Malvern Panalytical, Malvern, UK). One gram of each NCT-ME formulation was added to a beaker containing 100 mL of water followed by gentle stirring with a magnetic stirrer at 100 rpm before analysis. The droplet size distribution was expressed as a PDI value, which was obtained from the software provided by the instrument. This PDI value is an indication of the width of the particle size distribution, ranging from 0 (monodispersed) to 1 (very broad distribution). Zeta potential was also recorded on the same equipment. The data was obtained from three independent samples, each of which involved 12 individual tests.

The morphology of the dispersed droplets was investigated using TEM, according to a previous report [27], with some modifications. In brief, the samples were prepared by diluting 1 g of NCT-ME with 100 mL of water and gently mixing them using a magnetic stirrer at 100 rpm. Subsequently, the sample was placed on copper grids (200 mesh), then the dried sample was coated with 1% phosphoric acid and dried at 25 °C overnight. After that, the grid was loaded into a TEM sample holder. The droplet morphology was observed and recorded using a JEM 2200FS TEM (JEOL Co., Tokyo, Japan) with an energy filter installed (Omega filter, JEOL Co., Tokyo, Japan) and was operated at 200 kV. Digitized transmitted images were collected using a slow-scan CCD camera (Gatan USC1000, Gatan Inc., Pleasanton, CA, USA).

### 3.3. Preparation and Characterization of NCT-ME Fast-Dissolving Films

#### 3.3.1. NCT-ME Fast-Dissolving Films’ Preparation

The solvent casting technique was used to create the NCT-ME fast-dissolving films. Hydroalcoholic solution (ethanol: water = 9:1) of 5% *w*/*w* HPMC E15 was prepared by dispersing HPMC E15 in distilled water and stirring at room temperature (25 ± 2 °C) for 30 min. After that, 0.8% *w*/*w* NCT-ME was added to the HPMC E15 solution, was gently stirred for 5 min, and left until all the air bubbles had disappeared. Ten grams of the prepared solution was cast onto a Petri dish with a 9 cm diameter (the area of the Petri dish was 63.64 cm^2^) and was then dried overnight at room temperature. The obtained film was cut into a square shape with a size of 2 × 2 cm, containing approximately 2 mg of NCT. NCT films without ME (NCT50-Smix0 film) were prepared and characterized to compare them with NCT-ME fast-dissolving films.

#### 3.3.2. Characterization of NCT-ME Fast-Dissolving Films

(a) Morphology Characterization of Films

Scanning electron microscopy (SEM, JEOL JSM-6610LV, Tokyo, Japan) was used to examine the morphology of NCT-ME fast-dissolving films. A square-shaped film with a size of 5 × 5 mm was cut to investigate the film’s surface, while a rectangle-shaped film with a size of about 1 × 5 mm was cut to investigate film thickness. The film was placed on a carbon tape, then the film surface was coated with gold for 15 s using a 40-mA sputter coater (JEOL JFC-1100E, Tokyo, Japan). The SEM images were collected at an accelerating voltage of 15 kV with 350× and 500× magnifications.

(b) Film Weight

Three films with 2 × 2 cm sizes were cut randomly from each film formulation. Films were weighed individually on an electronic balance (PA214, Ohaus Corporation, Parsippany, NJ, USA), and the mean weight was calculated.

(c) Film Thickness

The thickness of the film with a size of 2 × 2 cm was measured at three positions (left, center and right) in the same place on each film by an outside micrometer (3203-25A, Insize Co, Ltd., Suzhou, Jiangsu, China). The average film thickness (in mm) with standard deviations were calculated after five replicates for each film.

(d) In Vitro Disintegration Time Study

The disintegration test method of this study was adapted from Preis et al. [28]. The film was clamped in place by the sample holder (attached to the top side) and the magnetic clip (attached to the bottom side of the film). The magnetic clip weighed 3 g (0.03 N), which was roughly similar to the minimal force applied by the human tongue. The attached film was then half-immersed in 65 mL of simulated salivary fluid (SSF) pH 6.8 at 37 ± 0.5 °C. The SSF contained 8.0 g/L sodium chloride, 0.19 g/L potassium phosphate monobasic, and 2.98 g/L sodium phosphate dibasic dihydrate. Hydrochloric acid at 1 M was used to adjust the pH to 6.8 [29]. In vitro disintegration time was measured by visually recording the time required for the film to break and the magnetic clip to fall down. For each formulation, experiments were carried out in triplicate. The obtained disintegration times were normalized by the film thickness for direct comparisons of in vitro disintegration times.

(e) Mechanical Strength Test

The mechanical strength of the films was measured by a texture analyzer TX.TA plus (Stable Micro Systems, Surrey, UK). An individual sample holder has been constructed to facilitate measurements of a 2 × 2 cm sized film samples. The dry film was adhered to the plate with a 9.0 mm diameter cylindrical hole (area of the sample holder hole = 63.56 mm^2^). A cylindrical stainless probe (2 mm diameter) with a plane flat-faced surface was used (probe contact area = 3.14 mm^2^). The texture analyzer was set for the probe’s forward movement at a velocity of 1.0 mm/s. Measurement started when the probe had contacted the sample surface (triggering force). The probe continued to advance at a consistent speed until the film was ripped apart. The applied force and distance were recorded. All of the experiments were conducted at a room temperature (25 ± 2 °C, 70% relative humidity). Each film was subjected to five replications. The mechanical strength of the film was characterized by tensile strength, normalize tensile strength, elongation at break, and Young’s modulus [30,31,32].

### 3.4. NCT Loading Efficiency

Three randomly selected films (size of 2 × 2 cm) were added to vials containing 10 mL of 50% *v*/*v* ethanol and were gently stirred for 15 min, or until the film was absolutely dissolved. The solution was withdrawn, filtered, and diluted 2 times with 50% *v*/*v* ethanol before HPLC analysis. The HPLC condition was analyzed using an Agilent HP1100 HPLC instrument (Agilent Technologies, Santa Clara, CA, USA) with an auto sampler. The stationary phase used was a C-18 reverse-phase column, 4.6 × 150 mm (Agilent technologies, Santa Clara, CA, USA). As mobile phase, a mixture of sodium acetate solution, methanol, and trimethylamine at 88:12:0.5 *v*/*v*, pH 4.2 was used. The flow rate of column was 1 mL/min with UV detection at 259 nm [33]. The retention time of NCT was detected at approximately 3.2 min. The standard curve of NCT in 50% *v*/*v* ethanol, which had proven linearity, with a high correlation coefficient (R^2^ = 0.9999), was used to calculate NCT contents. The following regression equation was found: y = 3.8855x − 8.1691, where y is the absorbance and x is the concentration of NCT (µg/mL). The experiment was carried out in triplicate. The percentage of NCT loading efficiency in the NCT-ME fast-dissolving films was calculated using the following Equation (1).

(1) $$\mathrm{NCT loading efficiency}\;(\%)=\frac{\mathrm{Mass}\mathrm{of}\mathrm{NCT}\mathrm{in}\mathrm{film}}{\mathrm{Mass}\mathrm{of}\mathrm{NCT}\mathrm{in}\mathrm{film}\mathrm{feed}}\times 100\%$$

### 3.5. In Vitro NCT Release Study

NCT-ME films were placed into a beaker containing 20 mL of SSF pH 6.8 as a medium [34]. The beaker was placed over a magnetic stirrer and the temperature of the assembly was maintained at 37 ± 2 °C throughout the experiment. The rpm was kept constant at 50 for this experiment. Samples (1 mL) were withdrawn at definite time intervals and were replaced with equal amounts of fresh medium. The samples were evaluated using HPLC after two times dilutions with ethanol, as described in procedure 3.4.

The drug release kinetics and mechanism were described using a variety of kinetic models. To receive the best-fit model, the in vitro dissolution data were correlated to the zero-order model, first-order model, Higuchi model, and Korsmeyer–Peppas empirical power law [25,35,36].

### 3.6. Statistical Analysis

The statistical evaluation of mean droplet size and size distribution of MEs, as well as the characterization of NCT-ME fast-dissolving films were performed by ANOVA using SPSS software version 17.0 (SPSS Inc., Chicago, IL, USA). Data are presented as mean ± SD and *p* < 0.05 was considered to indicate significant differences.

## 4. Conclusions

This research aimed to develop NCT-ME fast-dissolving films using HPMC as a film-forming agent for the buccal film. NCT-ME formulations were first developed with less than 100 nm mean droplet sizes and a narrow size distribution. The obtained zeta potential represents the negatively charged surface caused by negatively charged ions of the NCT. The different compositions of NCT-ME presented no significant differences in droplet size and zeta potential. The ratio between surfactant and co-surfactant and type of surfactant did not affect the ME formulation except for NCT40-Smix[P-80(1:2)]10. NCT-ME formulations containing the most NCT and which yielded particle sizes of less than 100 nm, with narrow PDI values, were NCT40-Smix[PEG-40H(1:2)]10, NCT40-Smix[P-80(1:1)]10, NCT40-Smix[PEG-40H(1:1)]10, NCT40-Smix[P-80(2:1)]10, and NCT40-Smix[PEG-40H(2:1)]10. These formulations were chosen to be incorporated into fast-dissolving films. After 100-fold dilutions of selected NCT-ME formulations, TEM images presented small droplet sizes with a spherical form. After that, the selected NCT-ME was incorporated into a fast-dissolving film by a solvent casting process. The obtained NCT-ME fast-dissolving films had a uniformity of NCT throughout the film formulation. The normalized disintegration time of the NCT-ME fast-dissolving films was less than 32 s, which is an acceptable disintegration time for a fast-dissolving film. The NCT-ME incorporated in the film significantly influenced the normalized tensile strength, percent elongation at break, and Young’s modulus values. The NCT40-Smix[PEG-40H(2:1)]10 film had a lower normalized tensile strength and percent elongation at break than NCT50-Smix0 films (surfactant- and co-surfactant-free formulation), leading to the shortest disintegration time. The NCT40-Smix[PEG-40H(1:2)]10 film and NCT40-Smix[PEG-40H(2:1)]10 film had decreased normalized tensile strength and percent elongation at break when compared to the NCT50-Smix0 film. All NCT-ME films except for NCT40-Smix[PEG-40H(2:1)]10 film could alternate Young’s modulus values when compared to the NCT50-Smix0 film. An ideal buccal film should have a sufficiently high tensile strength and high Young’s modulus values to endure regular handling. The NCT-ME fast-dissolving films exhibited very high NCT loading contents, more than 100%. According to the drug release profile of NCT-ME fast-dissolving films, NCT was released rapidly in the first 3 min. The drug release kinetic of all NCT-ME fast-dissolving films were fitted with the Higuchi model, which describes that drug release is regulated by Fickian diffusion. It was concluded that the developed films with ME can increase the dissolution rate profile.

## Figures and Tables

**Table 1:** The mean droplet size, size distribution expressed as PDI values, and zeta potential of each NCT-ME.

Formulation	Droplet Size (nm)	PDI Value	Zeta Potential (mV)
NCT50-Smix0	1237 ± 39.66 ^a^	0.73 ± 0.0 ^a^	−73.7 ± 4.1 ^a^
Smix weight ratio of 1:2			
NCT40-Smix[P-80(1:2)]10	215.8 ± 59.9 ^b^	0.34 ± 0.10 ^b^	−25.5 ± 0.5 ^b^
NCT30-Smix[P-80(1:2)]20	18.4 ± 12.6 ^c^	0.29 ± 0.05 ^b^	−20.4 ± 4.8 ^b^
NCT20-Smix[P-80(1:2)]30	69.7 ± 43.4 ^c^	0.19 ± 0.07 ^b^	−13.4 ± 3.2 ^b^
NCT10-Smix[P-80(1:2)]40	15.5 ± 0.2 ^c^	0.25 ± 0.02 ^b^	−11.4 ± 0.9 ^b^
NCT40-Smix[PEG-40H(1:2)]10	21.7 ± 0.1 ^c^	0.26 ± 0.02 ^b^	−24.5 ± 6.1 ^b^
NCT30-Smix[PEG-40H (1:2)]20	15.1 ± 0.8 ^c^	0.28 ± 0.02 ^b^	−10.9 ± 1.6 ^b^
NCT20-Smix[PEG-40H (1:2)]30	21.1 ± 13.6 ^c^	0.21 ± 0.04 ^b^	−10.9 ± 3.6 ^b^
NCT10-Smix[PEG-40H (1:2)]40	12.1 ± 0.6 ^c^	0.17 ± 0.04 ^b^	−10.8 ± 1.6 ^b^
Smix weight ratio of 1:1			
NCT40-Smix[P-80(1:1)]10	41.9 ± 10.6 ^c^	0.31 ± 0.10 ^b^	−16.4 ± 10.9 ^b^
NCT30-Smix[P-80(1:1)]20	19.8 ± 5.0 ^c^	0.21 ± 0.10 ^b^	−13.6 ± 2.1 ^b^
NCT20-Smix[P-80(1:1)]30	38.9 ± 15.4 ^c^	0.29 ± 0.10 ^b^	−11.3 ± 2.2 ^b^
NCT10-Smix[P-80(1:1)]40	63.1 ± 48.4 ^c^	0.25 ± 0.05 ^b^	−18.7 ± 5.0 ^b^
NCT40-Smix[PEG-40H(1:1)]10	24.3 ± 0.2 ^c^	0.30 ± 0.10 ^b^	−14.5 ± 5.4 ^b^
NCT30-Smix[PEG-40H (1:1)]20	20.4 ± 0.1 ^c^	0.30 ± 0.0 ^b^	−21.6 ± 6.3 ^b^
NCT20-Smix[PEG-40H (1:1)]30	18.5 ± 0.4 ^c^	0.27 ± 0.0 ^b^	−15.9 ± 0.3 ^b^
NCT10-Smix[PEG-40H (1:1)]40	18.5 ± 0.3 ^c^	0.23 ± 0.0 ^b^	−15.0 ± 4.1 ^b^
Smix weight ratio of 2:1			
NCT40-Smix[P-80(2:1)]10	33.0 ± 18.0 ^c^	0.24 ± 0.03 ^b^	−18.4 ± 5.1 ^b^
NCT30-Smix[P-80(2:1)]20	21.9 ± 7.4 ^c^	0.19 ± 0.07 ^b^	−19.2 ± 5.3 ^b^
NCT20-Smix[P-80(2:1)]30	12.6 ± 0.4 ^c^	0.26 ± 0.03 ^b^	−17.1 ± 11.0 ^b^
NCT10-Smix[P-80(2:1)]40	44.6 ± 21.8 ^c^	0.21 ± 0.08 ^b^	−17.2 ± 2.3 ^b^
NCT40-Smix[PEG-40H(2:1)]10	28.5 ± 12.4 ^c^	0.25 ± 0.02 ^b^	−17.5 ± 5.6 ^b^
NCT30-Smix[PEG-40H (2:1)]20	19.5 ± 2.8 ^c^	0.27 ± 0.02 ^b^	−13.4 ± 3.0 ^b^
NCT20-Smix[PEG-40H (2:1)]30	19.6 ± 0.9 ^c^	0.26 ± 0.04 ^b^	−11.6 ± 1.8 ^b^
NCT10-Smix[PEG-40H (2:1)]40	39.8 ± 36.4 ^c^	0.19 ± 0.07 ^b^	−10.6 ± 3.0 ^b^

Note: For each test, means with the same letter are not significantly different. Thus, means with different letters, e.g., “^a^”, “^b^” or “^c^”, are statistically different (*p* < 0.05).

**Table 2:** Weight, thickness, and in vitro disintegration time of the selected NCT-ME fast-dissolving films.

Formulation	Weight (mg ± SD)	Thickness (mm ± SD)	Disintegration Time (s)	Normalized Disintegration Time (s)
NCT50-Smix0 film	36.77 ± 5.56 ^a^	0.083 ± 0.008 ^a^	28.83 ± 8.28 ^a^	25.27 ± 5.12 ^a^
NCT40-Smix[PEG-40H(1:2)]10 film	39.07 ± 3.80 ^a^	0.079 ± 0.003 ^a^	28.71 ± 11.58 ^a^	26.08 ± 9.53 ^a^
NCT40-Smix[P-80(1:1)]10 film	39.67 ± 5.24 ^a^	0.085 ± 0.006 ^a^	28.95 ± 8.85 ^a^	24.45 ± 6.80 ^a^
NCT40-Smix[PEG-40H(1:1)]10 film	40.90 ± 1.31 ^a^	0.080 ± 0.003 ^a^	32.91 ± 8.64 ^a^	29.58 ± 7.05 ^a^
NCT40-Smix[P-80(2:1)]10 film	36.03 ± 1.84 ^a^	0.073 ± 0.003 ^a^	31.22 ± 6.28 ^a^	31.10 ± 5.12 ^a^
NCT40-Smix[PEG-40H(2:1)]10 film	39.13 ± 6.44 ^a^	0.085 ± 0.015 ^a^	16.04 ± 3.94 ^b^	14.22 ± 2.32 ^b^

Note: For each test, means with the same letter are not significantly different. Thus, means with different letters, e.g., “^a^” or “^b^”, are statistically different (*p* < 0.05).

**Table 3:** Mechanical characteristics of the selected NCT-ME fast-dissolving films.

Formulation	Tensile Strength (MPa)	Normalized Tensile Strength (MPa)	Elongation at Break (%)	Young’s Modulus (MPa)	NCT Loading Efficiency (%)
NCT50-Smix0 film	6.31 ± 0.46 ^a^	5.51 ± 0.18 ^a^	17.77 ± 0.39 ^a^	93.91 ± 3.14 ^a^	110.69 ± 2.17 ^a^
NCT40-Smix[PEG-40H(1:2)]10 film	5.55 ± 0.22 ^a^	5.09 ± 0.16 ^b^	12.61 ± 1.78 ^b^	103.35 ± 5.72 ^b^	112.11 ± 2.59 ^a^
NCT40-Smix[P-80(1:1)]10 film	5.63 ± 0.87 ^a^	4.78 ± 0.70 ^b^	20.82 ± 3.61 ^a^	77.68 ± 11.06 ^c^	115.47 ± 4.21 ^b^
NCT40-Smix[PEG-40H(1:1)]10 film	5.99 ± 0.39 ^a^	5.41 ± 0.26 ^a^	19.63 ± 2.82 ^a^	88.17 ± 5.73 ^c^	103.08 ± 3.95 ^c^
NCT40-Smix[P-80(2:1)]10 film	5.82 ± 0.07 ^a^	5.82 ± 0.15 ^a^	12.60 ± 0.30 ^b^	115.54 ± 3.85 ^b^	115.30 ± 5.68 ^b^
NCT40-Smix[PEG-40H(2:1)]10 film	5.79 ± 0.93 ^a^	4.96 ± 0.49 ^b^	15.02 ± 4.11 ^c^	92.79 ± 10.73 ^a^	107.73 ± 2.87 ^a^

Note: For each test, means with the same letter are not significantly different. Thus, means with different letters, e.g., “^a^”, “^b^” or “^c^”, are statistically different (*p* < 0.05).

**Table 4:** Release kinetic data of NCT-ME fast-dissolving films using various kinetic models.

Kinetic Model	Parameter	NCT-ME Fast-Dissolving Films				
		NCT50-Smix0 Film	NCT40-Smix[PEG-40H(1:2)]10 Film	NCT40-Smix[P-80(1:1)]10 Film	NCT40-Smix[P-80(2:1)]10 Film	NCT40-Smix[PEG-40H(2:1)]10 Film
Zero-order	*r^2^*	0.5388	0.5226	0.4522	0.4774	0.5154
	*k*_0_ (min^−1^)	2.9442	2.9599	2.7975	2.7496	2.8401
First-order	*r^2^*	0.4250	0.4094	0.3341	0.3234	0.3580
	*k*_1_ (min^−1^)	0.0214	0.0211	0.0197	0.0190	0.0201
Higuchi	*r^2^*	0.9750	0.9669	0.9734	0.9319	0.8618
	*k*_H_ (min^1/2^)	50.832	48.487	60.602	52.930	44.227
Korsmeyer–Peppas	*r^2^*	0.9661	0.9586	0.9454	0.8735	0.8611
	*k* (min^−n^)	1.7434	1.6202	1.6726	1.6833	1.6454
	n	0.3472	0.6585	0.7714	0.7430	0.6990

**Table 5:** Type and concentration (% *w*/*w*) of the components used in each preparation of NCT-ME.

Formulation	Component (% *w/w*)				
	NCT	P-80	PEG-40H	PEG-400	Water
NCT50-Smix0	50				50
Smix weight ratio of 1:2					50
NCT40-Smix[P-80(1:2)]10	40	3.33		6.67	
NCT30-Smix[P-80(1:2)]20	30	6.67		13.33	
NCT20-Smix[P-80(1:2)]30	20	10		20	
NCT10-Smix[P-80(1:2)]40	10	13.33		26.67	
NCT40-Smix[PEG-40H(1:2)]10	40		3.33	6.67	
NCT30-Smix[PEG-40H (1:2)]20	30		6.67	13.33	
NCT20-Smix[PEG-40H (1:2)]30	20		10	20	
NCT10-Smix[PEG-40H (1:2)]40	10		13.33	26.67	
Smix weight ratio of 1:1					50
NCT40-Smix[P-80(1:1)]10	40	5		5	
NCT30-Smix[P-80(1:1)]20	30	10		10	
NCT20-Smix[P-80(1:1)]30	20	15		15	
NCT10-Smix[P-80(1:1)]40	10	20		20	
NCT40-Smix[PEG-40H(1:1)]10	40		5	5	
NCT30-Smix[PEG-40H (1:1)]20	30		10	10	
NCT20-Smix[PEG-40H (1:1)]30	20		15	15	
NCT10-Smix[PEG-40H (1:1)]40	10		20	20	
Smix weight ratio of 2:1					
NCT40-Smix[P-80(2:1)]10	40	6.67		3.33	
NCT30-Smix[P-80(2:1)]20	30	13.33		6.67	
NCT20-Smix[P-80(2:1)]30	20	20		10	
NCT10-Smix[P-80(2:1)]40	10	26.67		13.33	50
NCT40-Smix[PEG-40H(2:1)]10	40		6.67	3.33	
NCT30-Smix[PEG-40H (2:1)]20	30		13.33	6.67	
NCT20-Smix[PEG-40H (2:1)]30	20		20	10	
NCT10-Smix[PEG-40H (2:1)]40	10		26.67	13.33	

## Data Availability

Not applicable.

## Sample Availability

Samples of the compounds are obtained from commercially available company.
